# Cardiovascular health predicts working memory decline among cognitively healthy older African Americans

**DOI:** 10.1002/alz.095802

**Published:** 2025-01-09

**Authors:** Brad Amoateng, Mustafa Sheikh, Bernadette A. Fausto, Mark A. Gluck

**Affiliations:** ^1^ Rutgers University–Newark, Newark, NJ USA

## Abstract

**Background:**

African Americans are at increased risk for cardiovascular‐related health problems (e.g., hypertension, cardiovascular disease, stroke, obesity) and cognitive dysfunction, including Alzheimer’s disease (AD)^1,2^. However, few studies have looked at the longitudinal influence of cardiovascular health and cognitive function in older African Americans. This study investigated the relationship between cardiovascular health markers and cognition in older African Americans.

**Method:**

836 participants were drawn from Pathways to Healthy Aging in African Americans, a longitudinal cohort study at Rutgers University–Newark. Participants completed a battery of self‐reported health and demographic questionnaires, neuropsychological tests, and a short physical battery of both anthropometric and physical assessments. Linear mixed models were used to examine the relationship between baseline cardiovascular health markers and cognitive function up to 6 years of follow up.

**Result:**

Higher diastolic blood pressure was significantly associated with worse Trail Making Test Part A (B = 0.035, p < .001), and Montreal Cognitive Assessment (B = 0.085, p = .0029) performance across time. Higher systolic blood pressure was significantly associated with poorer Trail Making Test Part A (B = 0.017, p < .001) and MoCA (B = 0.055, p < .001). Higher resting heart rate was significantly associated with worse Trail Making Test Part A (B = 0.018, p = .0031) and Part B (B = 0.085, p = .013), Controlled Oral Word Association Test (B = 0.036, p = .025), and Montreal Cognitive Assessment performance across time (B = 0.047, p = .046).

**Conclusion:**

In cognitively unimpaired older African Americans, cardiovascular health markers are predictive biomarkers for early working memory decline in preclinical AD. Our findings may inform holistic clinical decision‐making in AD prevention and personalized AD monitoring in patients with cardiovascular disease.

